# Chapter 3: Small Molecules and Disease

**DOI:** 10.1371/journal.pcbi.1002805

**Published:** 2012-12-27

**Authors:** David S. Wishart

**Affiliations:** 1 Department of Biological Sciences, University of Alberta, Edmonton, Alberta, Canada; 2 Department of Computing Science, University of Alberta, Edmonton, Alberta, Canada; 3 National Research Council, National Institute for Nanotechnology (NINT), Edmonton, Alberta, Canada; Whitehead Institute, United States of America; University of Maryland, Baltimore County, United States of America

## Abstract

“Big” molecules such as proteins and genes still continue to capture the imagination of most biologists, biochemists and bioinformaticians. “Small” molecules, on the other hand, are the molecules that most biologists, biochemists and bioinformaticians prefer to ignore. However, it is becoming increasingly apparent that small molecules such as amino acids, lipids and sugars play a far more important role in all aspects of disease etiology and disease treatment than we realized. This particular chapter focuses on an emerging field of bioinformatics called “chemical bioinformatics” – a discipline that has evolved to help address the blended chemical and molecular biological needs of toxicogenomics, pharmacogenomics, metabolomics and systems biology. In the following pages we will cover several topics related to chemical bioinformatics. First, a brief overview of some of the most important or useful chemical bioinformatic resources will be given. Second, a more detailed overview will be given on those particular resources that allow researchers to connect small molecules to diseases. This section will focus on describing a number of recently developed databases or knowledgebases that explicitly relate small molecules – either as the treatment, symptom or cause – to disease. Finally a short discussion will be provided on newly emerging software tools that exploit these databases as a means to discover new biomarkers or even new treatments for disease.

**What to Learn in This Chapter**

- The meaning of chemical bioinformatics
- Strengths and limitations of existing chemical bioinformatic databases
- Using databases to learn about the cause and treatment of diseases
- The Small Molecule Pathway Database (SMPDB)
- The Human Metabolome Database (HMDB)
- DrugBank
- The Toxin and Toxin-Target Database (T3DB)
- PolySearch and Metabolite Set Enrichment Analysis

This article is part of the “Translational Bioinformatics” collection for *PLOS Computational Biology*.

## 1. Introduction

For most of the past 100 years, the fields of toxicology, pharmacology and clinical biochemistry have focused on identifying the chemicals that cause (toxins), cure (drugs) or characterize (biomarkers) most human diseases. Historically, this kind of work has been reliant on the slow, careful and sometime tedious approaches of classical analytical chemistry and classical biochemistry. Nevertheless, it has led to important discoveries and enormous advances in our understanding of the actions of chemicals on genes, proteins and cells. With the recent emergence of high throughput “omics” technologies, our ability to detect, identify, and characterize small molecules along with their large molecule targets has been radically changed [1], [2]. Now it is possible to perform as many sequencing experiments, mass spectrometry (MS) experiments or compound identifications in a single day as used to be done in a single year. As a result, traditional fields such as toxicology, pharmacology and biochemistry have been transformed into totally new fields called toxicogenomics, pharmacogenomics and metabolomics. This transformation has changed not only the fundamentals of these disciplines, but also the fundamentals of their data. Rather than trying to manage a few samples, a few sequences or a few compounds in a paper notebook or on an Excel spreadsheet, researchers are confronted with the task of handling hundreds of samples, thousands of compounds, thousands of spectra and thousands of genes or protein sequences. This has led to the development of novel computational tools and entirely new bioinformatic disciplines to facilitate the handling of this data. This particular chapter focuses on an emerging field of bioinformatics called “chemical bioinformatics” – a discipline that has evolved to help address the blended chemical and molecular biological needs of toxicogenomics, pharmacogenomics, metabolomics and systems biology.

Chemical bioinformatics combines the sequence-centric tools of bioinformatics with the chemo-centric tools of “cheminformatics”. The term cheminformatics, which is an abbreviated form of “chemical informatics”, was first coined by Frank Brown nearly 15 years ago [3]. Cheminformatics (as it is known in North America) or chemoinformatics (as it is known in Europe and the rest of the world) is actually a close cousin to bioinformatics. Just as bioinformatics is a field of information technology concerned with using computers to analyze molecular biological data, cheminformatics is a field of information technology that uses computers to facilitate the collection, storage, analysis and manipulation of large quantities of chemical data.

However, there are some distinct “cultural” differences between bioinformatics and cheminformatics. For instance, cheminformatics software is mostly designed for use by chemists, while bioinformatics software is designed for use by molecular biologists. Consequently there is often a terminology gap that makes it difficult for biologists to use cheminformatic software and chemists to use bioinformatics software. Likewise, most cheminformatic software is structure-based or picture-driven while most bioinformatic software is sequence-based or text-driven. As a result, different search and query interfaces have evolved that are quite specific to either cheminformatic or bioinformatic software. Further compounding this culture gap is the fact that most cheminformatics software and chemical compound databases were developed without the expectation that this information would ever be biologically or medically relevant. Likewise, most bioinformatics software and bioinformatic databases were developed without the intention of using this data to facilitate small molecule biomarker identification or small molecule drug discovery. Consequently most biological sequence data is not linked in any meaningful way to drug or disease information – and vice versa. However, thanks to the emergence of new fields such as pharmacogenomics, toxicogenomics, systems biology and metabolomics, there is now a growing desire to bring bioinformatics and cheminformatics closer together. This has spawned the new field of chemical bioinformatics.

In this chapter we will cover several topics related to chemical bioinformatics. First, a brief overview of some of the most important chemical bioinformatic resources will be given. This will include a discussion of some of the major databases and classes of databases. Second, a more detailed overview will be given on those particular resources that allow researchers to connect small molecules to diseases. This section will focus on describing a number of recently developed databases or knowledgebases that explicitly relate small molecules – either as the treatment, symptom or cause – to disease. Finally a short discussion will be provided on newly emerging software tools that exploit these databases as a means to discover new biomarkers or even new treatments for disease.

## 2. Databases for Chemical Bioinformatics

Electronic databases lie at the heart of almost any subdiscipline of bioinformatics – and chemical bioinformatics is no exception. Indeed, without databases there is essentially no foundational knowledge to the discipline, and consequently, no compelling reason to write software. Programs such as BLAST [4] would be useless without GenBank [5], likewise, PSIPRED [6] couldn't exist without the Protein Databank [7] and Gene Set Enrichment Analysis – GSEA [8] would be impossible without the GEO and KEGG databases [9], [10]. Given their importance, it is perhaps worthwhile to briefly review the different types of chemical-bioinformatic databases that are available and discuss some of their particular strengths and limitations.

Currently there are four major classes of chemical-bioinformatic databases. These include: 1) small molecule (or metabolic) pathway databases; 2) metabolite or metabolomic databases; 3) drug databases; and 4) toxin or toxic substance databases. In an ideal world each of these database classes could/should be useful for relating small molecules to human diseases or disease treatments. For instance, metabolic pathway databases would be expected to be most useful for understanding the “big-picture” relationship between small molecules and disease – either with regard to those small molecule compounds causing disease (i.e. toxins), indicating disease (i.e. biomarkers) or being used in the treatment of disease (i.e. drugs). On the other hand, metabolite or metabolomic databases would be expected to be most useful for associating small molecule biomarkers with specific diseases, such as inborn errors of metabolism or a variety of chronic or infectious diseases characterized by metabolite imbalances. Drug databases would obviously be most relevant for identifying small molecules with disease treatments, although they could also be used to identify small molecule drugs causing adverse drug reactions. Finally toxin or toxic compound databases would be expected to be most useful for identifying the compounds causing diseases or causing symptoms associated with certain poisoning or environmental exposure incidents. This could include acute poisonings or more long-term, environmentally influenced conditions such as cancer, allergies or birth defects.

However, as detailed below, not all of the available chemical-bioinformatic databases are particularly suited for these kinds of disease-associated queries. This likely reflects the relatively nascent stage of this field (it's less than five years old) and the fact that disease-related information is much more difficult to gather and codify than either chemical structure or gene sequence information. Certainly all of today's existing chemical-bioinformatic databases contain information about different classes of chemicals (metabolites, drugs or poisons) and most contain some limited information about the corresponding protein and/or genetic targets. However, only a very small number of these databases actually include information on the diseases or physiological effects that may be caused, cured or characterized by these chemicals.

### 2.1 Metabolic Pathway Databases

Among the four major classes of chemical-bioinformatic databases that are available, metabolic pathway databases are perhaps the best known and most widely used. They include a number of popular web-based resources such as the Kyoto Encyclopedia of Genes and Genomes – also known as KEGG [10], the “Cyc” databases [11], [12], the Reactome database [13], WikiPathways [14], the Small Molecule Pathway Databases or SMPDB [15] and the Medical Biochemistry Page [http://themedicalbiochemistrypage.org/]. Several commercial pathway databases also exist such as TransPath (from BioBase Inc.), PathArt (from Jubilant Biosys Inc.), MetaBase (from GeneGo Inc.) and Ingenuity Pathways Analysis (Ingenuity Systems Inc.), many of which provide nicely illustrated metabolic pathway diagrams. Most of these pathway databases were designed to facilitate the exploration of metabolism and metabolites across many different species. This broad, multi-organism perspective has been critical to enhancing our basic understanding of metabolism and our appreciation of biological diversity. Metabolic pathway databases also serve as the backbone to facilitate many practical applications in biology including comparative genomics and targeted genome annotation. Table 1 lists the names, web addresses and general features for these and other useful pathway databases.

**Table 1:** Alphabetical List of Popular Metabolic Pathway Databases.

Database Name	URL or Web Address	Comments
HumanCyc (Encylopedia of Human Metabolic Pathways)	http://humancyc.org/	-MetaCyc adopted to human metabolism-No disease or drug pathways
KEGG (Kyoto Encyclopedia of Genes and Genomes)	http://www.genome.jp/kegg/	-Best known and among the most complete metabolic pathway databases-Covers many organisms-A Few disease and drug pathways
The Medical Biochemistry Page	http://themedicalbiochemistrypage.org/	-Simple metabolic pathway diagrams with extensive explanations-A few drug and disease pathways
MetaCyc (Encyclopedia of Metabolic Pathways)	http://metacyc.org/	-Similar to KEGG in coverage, but different emphasis-Well referenced-No disease or drug pathways
Reactome (A Curated Knowledgebase of Pathways)	http://www.reactome.org/	-Pathway database with more advanced query features-Not as complete as KEGG or MetaCyc
Roche Applied Sciences Biochemical Pathways Chart	http://www.expasy.org/cgi-bin/search-biochem-index	-The old metabolism standard (on line)-Describes most human metabolism
Small Molecule Pathway Database (SMPDB)	http://www.smpdb.ca/	-Pathway database with disease, drug and metabolic pathways for humans-Extensive search, analysis and visualization tools
Wikipathways	http://www.wikipathways.org	-Community annotated pathway database for 19 model organisms-Contains 175 human pathways-Few drug or disease pathways

Those metabolic pathway databases that strive for very broad organism coverage, such as KEGG and Reactome, tend to use pathway diagrams that are very generic and highly schematic, while those that are organism-specific (i.e. human-only), such as SMPDB and the Medical Biochemistry Page, tend to use diagrams that are very specific and much richer in detail, colour and content. Most pathway databases support interactive image mapping with hyperlinked information content that allows users to view chemical information (if a compound is clicked) or brief summaries of genes and/or proteins (if a protein is clicked). Almost all of the databases support some kind of limited text search and a few, such as Reactome, SMPDB and the “Cyc” databases, support the mapping of gene, protein and/or metabolite expression data onto pathway diagrams. As might be expected, the major focus of most of today's small molecule pathway databases is on basic metabolism. As a result, only one of these databases (SMPD) actually includes any pathways associated with drug action or disease.

### 2.2 Metabolomic Databases

The second major class of chemical-bioinformatic databases are metabolomic or metabolite databases. These databases tend to have a major focus on chemicals and chemical descriptors with a lesser (or even absent) focus on biological data. They are primarily used for metabolite identification – especially in metabolomic studies. Some databases are almost exclusively chemical in nature, containing primarily information on the chemical name(s), synonyms, InChI (International Chemical Identifier) identifier, structure, and molecular weight. These include Lipid Maps [16], a comprehensive database of biological lipids; ChEBI [17], a database of biologically interesting compounds; PubChem [18], a collection of most known organic chemicals with links to PubMed articles and more than 500,000 bioassays; ChemSpider [19], a chemical databases that is similar in size to PubChem; KNApSAcK [20], a database of plant phytochemicals and METLIN [21], a database of known and presumptive human metabolites. All of these databases support a variety of text search options and a few (such as PubChem, ChemSpider, LipidMaps and ChEBI) support structure and structure similarity searches. In addition to these biochemical databases, there are a number of smaller databases that contain spectral (NMR or MS) data of small molecule metabolites. These include the BioMagResBank or BMRB [22] which contains experimental NMR spectra of mammalian metabolites, MassBank [23] which contains MS spectra of a variety of metabolites, drugs and toxic compounds, MMCD [24] which contains experimental and predicted NMR spectra of *Arabadopsis* metabolites, and the Golm Metabolome database [25] which contains MS spectra of different plant metabolites. These spectral databases are frequently used to facilitate compound identification via spectral comparison. More recently, a much more comprehensive kind of metabolomic database has emerged which attempts to combine chemical data, spectral data, protein target data, biomarker data and disease data into a single resource. Perhaps the best example of this is the Human Metabolome Database (HMDB). The HMDB is a database containing comprehensive data on most of the known or measurable endogenous metabolites in humans [26]. Table 2 presents a summary of the names, web addresses and general features for the major metabolite/metabolomic databases.

**Table 2:** Alphabetical List of Metabolomic, Chemical or Spectral Databases.

Database Name	URL or Web Address	Comments
BioMagResBank (BMRB – Metabolimics)	http://www.bmrb.wisc.edu/metabolomics/	-Emphasis on NMR data, no biological or biochemical data-Specific to plants (Arabadopsis)
Chemicals Entities of Biological Interest (ChEBI)	http://www.ebi.ac.uk/chebi/	-Covers metabolites and drugs of biological interest-Focus on ontology and nomenclature not biology
ChemSpider	http://www.chemspider.com/	-Meta-database containing chemical data from 100+ other databases-20+ million compounds-Good search utilities
Golm Metabolome Database	http://csbdb.mpimp-golm.mpg.de/csbdb/gmd/gmd.html	-Emphasis on MS or GC-MS data only-No biological data-Few data fields-Specific to plants
Human Metabolome Database	http://www.hmdb.ca	-Largest and most completely annotated metabolomic database-Specific to humans only
KNApSAcK	http://kanaya.naist.jp/KNApSAcK/	-A phytochemical database containing data for 50,000 compounds
LipidMaps	http://www.lipidmaps.org/	-Contains 22,500 different lipids found in plants & animals-Nomenclature standard
METLIN Metabolite Database	http://metlin.scripps.edu/	-Human specific metabolite database-Name, structure, ID only
PubChem	http://pubchem.ncbi.nlm.nih.gov/	-Database containing 27 million unique chemicals with links to Bioassays and PubMed abstracts

### 2.3 Pharmaceutical Product Databases

The third major class of chemical bioinformatic databases are the drug or pharmaceutical product databases. In particular, two types of electronic drug databases have started to emerge over the past five years: 1) clinically oriented drug databases and 2) chemically oriented drug databases. Examples of some of the better-known clinically oriented drug databases include DailyMed [27] and RxList [28]. These resources typically offer very detailed clinical information (i.e. their formulation, metabolism and indications) about selected drugs derived from their FDA labels. As a result, these kinds of databases are targeted more towards pharmacists, physicians or consumers. Examples of chemically or genetically oriented drug databases include the TTD [29], PharmGKB [30] and SuperTarget [31]. TTD (which stands for Therapeutic Target Database) contains information on 5028 drugs (both approved and experimental) with 1894 identified targets and links to 560 different diseases or indications. PharmGKB (which stands for Pharmacogenomics Knowldege Base) has information on 1587 approved drugs (with descriptions and indications), including pharmacogenomic data on 287 drugs. SuperTarget contains information on more than 2500 target proteins, which are annotated with about 7300 literature-mined relations to 1500 different drugs. All three of these databases provide synoptic data (5–10 data fields per entry) about the nomenclature, structure and/or physical properties of small molecule drugs and, in the case of SuperTarget and TTD, their drug targets. Both TTD and SuperTarget support text, sequence and chemical structure searches, while PharmGKB provides mechanistic, pharmacodynamic and pharmacokinetic pathway information for 68 different drugs or drug classes. As a general rule, chemically oriented drug databases tend to appeal to medicinal chemists, biochemists and molecular biologists. In addition to these somewhat specialized databases, a much more comprehensive “hybrid” database, known as DrugBank [32] has recently been developed. Drugbank combines the clinical/disease information of the clinically oriented drug databases with the biochemical/chemical information of the chemically oriented drug databases. As a result, a typical DrugBank entry contains 80–100 different data fields, instead of 5–10 as seen with the other kinds of databases. Like TTD and SuperTarget, DrugBank supports very extensive text, sequence and chemical structure searches. It also provides detailed pathway information on the mechanism of action for >200 different drugs or drug classes. Table 3 provides a short summary of the names, descriptions and website addresses of the more popular drug or pharmaceutical product databases.

**Table 3:** Alphabetical List of Pharmaceutical Compound or Drug Databases.

Database Name	URL or Web Address	Comments
DailyMed	http://dailymed.nlm.nih.gov/	-A drug database containing FDA label (package inserts) for most approved drugs
DrugBank	http://www.drugbank.ca/	-Comprehensive database of 1480 drugs with 1700 drug targets-Contains chemical, biological & clinical data-Extensive search utilities
PharmGKB	http://www.pharmgkb.org/	-Data on 1587 approved drugs including pharmacogenomic data on 287 drugs.-Provides mechanistic, pathway information for 68 different drugs
SuperTarget	http://bioinf-tomcat.charite.de/supertarget/	-Searchable database of drugs and drug targets-Includes 2500 target proteins, which are annotated with about 7300 literature-mined relations to 1500 different drugs.
TTD (Therapeutic Target Database)	http://xin.cz3.nus.edu.sg/group/ttd/ttd.asp	-Contains data on 1894 drug targets for 5126 drugs-Limited chemical data-No clinical or pharmacological data

### 2.4 Toxic Substance Databases

The final class of chemical-bioinformatic databases we will discuss are the toxic substance databases. These include the Animal Toxin Database (ATDB), SuperToxic [33], ACToR [34], the Comparative Toxicogenomics Database [35] and T3DB [36]. Table 4 presents a summary of the names, web addresses and general features for these databases. The Animal Toxin Database (ATDB), with >3800 peptide toxins, provides data on the sequence of many peptide/protein toxins from venomous insects and animals as well as information on the channel targets to which these toxins bind. Both ACToR (which stands for the Aggregated Computational Toxicology Resource) and SuperToxic provide bioassy data and chemical structure information for a very large number of industrial or pharmaceutically interesting chemicals (>60,000 for SuperToxic, >500,000 for AcTOR). The Comparative Toxicogenomics Database (CTD), with >5000 chemicals, provides literature-derived information on chemical-gene interactions. This includes microarray information on genes that are up/down-regulated upon contact or exposure to these chemicals. T3DB (which stands for the Toxin, Toxin-Target Database) provides very extensive structural, physiological, mechanistic, medical and biochemical information on about 3100 commonly encountered (i.e. household or environmental) toxins and poisons.

**Table 4:** Alphabetical Listing of Toxic Compound Databases.

Database Name	URL or Web Address	Comments
ACToR (Aggregated Computation Toxicology Resource)	http://actor.epa.gov/actor/faces/ACToRHome.jsp	-Contains aggregated data on 2,500,000 environmental chemicals-Searchable by chemical name and structure-Data includes chemical structure, physico-chemical values, in vitro assay data and in vivo toxicology data.
ATDB (Animal Toxin Database)	http://protchem.hunnu.edu.cn/toxin/index.jsp	-Database with >3800 peptide toxins-Provides sequence data on peptide/protein toxins from venomous insects and animals
CTD (Comparative Toxicogenomic Database)	http://ctd.mdibl.org/	-Data on >5000 chemicals with literature-derived information on chemical-gene interactions
SuperToxic	http://bioinformatics.charite.de/supertoxic/	-Contains data on 60,000 toxic compounds and some target data-Provides chemical and toxicity information-Can predict the toxicity of query compounds
T3DB (Toxin, Toxin-Target Database)	http://www.t3db.org/	-Searchable database of 3100 common toxins and 1400 target proteins-Provides extensive structural, physiological, mechanistic, medical and biochemical information

Each of these databases addresses the needs of certain communities such as animal physiologists (ATDB), toxicogenomics or toxicology specialists (CTD and T3DB), environmental or industrial regulators (ACToR) or medicinal chemists interested in toxicity prediction (SuperToxic). However, with the exception of T3DB, most of these online toxin or toxic compound databases are relatively lightly annotated, with fewer than a dozen data fields per compound and essentially no physiological, disease or disease symptom information.

Clearly not all of the chemical-bioinformatic databases we have described in this section are suitable for deriving information about small molecules and disease. Likewise, many of the databases mentioned above are not exactly suitable for translational bioinformatic questions or for applications relating to medicine, medical biochemistry or clinical research. However, there is at least one database in each of the four major chemical-bioinformatic database classes that does generally meet these criteria. In particular: 1) SMPDB is a pathway database that explicitly relates small molecules to disease and disease treatment; 2) HMDB is a metabolomic database that associates metabolites to disease biomarkers or disease diagnosis; 3) DrugBank is a drug database that links drugs and drug targets to symptoms, diseases and disease treatments and 4) T3DB is a toxic substance database that associates toxins and their biological targets with symptoms, conditions, diseases and disease treatments. A more detailed description of each of these databases is provided below.

## 3. SMPDB – A Pathway Database for Drugs and Disease

**Figure 1:**
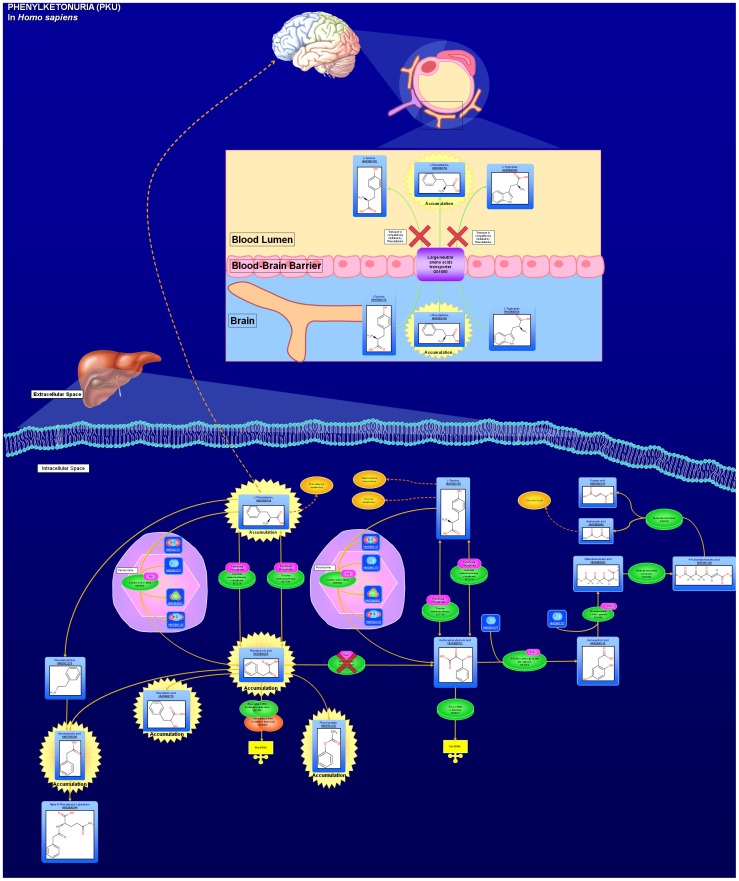
A pathway diagram for Phenylketonuria as taken from SMPDB (http://www.smpdb.ca).

As noted earlier, SMPDB is a pathway database specifically designed to facilitate clinical “omics” studies, with a specific emphasis on clinical biochemistry and clinical pharmacology. Currently SMPD consists of more than 450 highly detailed, hand-drawn pathways describing small molecule metabolism or small molecule processes that are specific to humans. These pathways can be placed into four different categories: 1) metabolic pathways; 2) small molecule disease pathways; 3) small molecule drug pathways and 4) small molecule signaling pathways. An example of a typical SMPDB pathway (Phenylketonuria) is shown in Figure 1. As seen in this figure, all SMPDB pathways explicitly include the chemical structure of the major chemicals in each pathway. In addition, the cellular locations (membrane, cytoplasm, mitochondrion, nucleus, peroxisome, etc.) of all metabolites and the enzymes involved in their processing are explicitly illustrated. Likewise the quaternary structures (if known) and cofactors associated with each of the pathway proteins are also shown. If some of the metabolic processes occur primarily in one organ or in the intestinal microflora, this information is also illustrated. The inclusion of explicit chemical, cellular and physiological information is one of the more unique and useful features of SMPDB. SMPDB is also unique in its inclusion of significant numbers of metabolic disease pathways (>100) and drug pathways (>200) not found in any other pathway database. Likewise, unlike other pathway databases, SMPDB supports a number of unique database querying and viewing features. These include simplified database browsing, the generation of protein/metabolite lists for each pathway, text querying, chemical structure querying and sequence querying, as well as large-scale pathway mapping via protein, gene or chemical compound lists.

The SMPDB interface is largely modeled after the interface used for DrugBank [32], T3DB [36] and the HMDB [26], with a navigation panel for Browsing, Searching and Downloading the database. Below the navigation panel is a simple text query box that supports general text queries of the entire textual content of the database. Mousing over the Browse button allows users to choose between two browsing options, SMP-BROWSE and SMP-TOC. SMP-TOC is a scrollable hyperlinked table of contents that lists all pathways by name and category. SMP-BROWSE is a more comprehensive browsing tool that provides a tabular synopsis of SMPDB's content with thumbnail images of the pathway diagrams, textual descriptions of the pathways, as well as lists of the corresponding chemical components and enzyme/protein components. This browse view allows users to scroll through the database, select different pathway categories or re-sort its contents. Clicking on a given thumbnail image or the SMPDB pathway button brings up a full-screen image for the corresponding pathway. Once “opened” the pathway image may be expanded by clicking on the Zoom button located at the top and bottom of the image. An image legend link is also available beside the Zoom button.

At the top of each pathway image is a pathway synopsis contained in a yellow box while at the bottom of each image is a list of references. On the right of each pathway image is a grey-green Highlight/Analyzer tool with a list of the key metabolites/drugs and enzymes/proteins found in the pathway. Checking on selected items when in the SMP-Highlight mode will cause the corresponding metabolite or protein in the pathway image to be highlighted with a red box. Entering concentration or relative expression values (arbitrary units) beside compound or protein names, when in the SMP-Analyzer mode, will cause the corresponding metabolites or proteins to be highlighted with differing shades of green or red to illustrate increased or decreased concentrations. As with most pathway databases, all of the chemical structures and proteins/enzymes illustrated in SMPDB's diagrams are hyperlinked to other on-line databases or tables. Specifically, all metabolites, drugs or proteins shown in the SMP-BROWSE tables or in a pathway diagram are linked to HMDB, DrugBank or UniProt [37] respectively. Therefore, clicking on a chemical or protein image will open a new browser window with the corresponding DrugCard, MetaboCard or UniProt table being displayed.

The most powerful search option in SMPDB is SMP-MAP, which offers both multi-identifier searches as well as “Omic” (transcriptomic, proteomic or metabolomic) mapping. In contrast to SMP-BROWSE, which is used for data browsing and single entity highlighting, SMP-MAP can be used for multi-entity highlighting and mapping. In particular SMP-MAP allows users to enter lists of chemical names, gene names, protein names, UniProt IDs, GenBank IDs, Agilent IDs or Affymetrix IDs (with or without concentration data) and to have a table generated of pathways containing those components. The resulting table, like the SMP-BROWSE table, displays a thumbnail image of the matching pathways along with the list of matching components (metabolites, drugs, proteins, etc.). The table is ordered by the number of matches and a significance score (calculated via a hypergeometric function), with the pathway having the most matches being placed at the top. Clicking on the thumbnail image or the SMPDB pathway button brings up a full-screen image for the corresponding pathway with all the matching components (metabolites, drugs, proteins, etc.) highlighted in red. Concentration data can be displayed using a red-to-yellow gradient by entering concentration data in a text box located beside the map image.

SMPDB's Search menu also offers users a choice of searching the database by chemical structure (ChemQuery), text (TextQuery) or sequence (SeqSearch). The ChemQuery option allows users to draw (using MarvinSketch applet) or write (using a SMILES string) a chemical compound and to search SMPDB for drugs and metabolites similar or identical to the query compound. The TextQuery button supports a more sophisticated text search (partial word matches, data field selection, Boolean text searches, case sensitive, misspellings, etc.) of the text portion of SMPDB, including the accompanying pathway explanations and reference sections. The SeqSearch button allows users to conduct BLASTP (protein) sequence searches of the protein sequences contained in SMPDB. SeqSearch supports both single and multiple sequence BLAST queries.

To summarize, SMPDB allows users to interactively explore, through detailed pathway diagrams, the linkage between metabolites, genes or proteins and metabolic diseases. It also allows users to investigate the connection between drugs and their protein or gene targets through comprehensive illustrations of their mechanism of action. Because of its detailed depictions of both disease and drug pathways and its extensive use of visualization and query tools, SMPDB can potentially support a variety of translational bionformatic/cheminformatic questions. For example, through SMPDB it is possible for users to: 1) identify a metabolic disease or medical condition given a list of metabolites (via SMP-MAP); 2) use experimental gene expression data to identify which diseases, conditions or pathways are most affected by a given drug, dietary or chemical treatment (via SMP-MAP); 3) use metabolomic or metabolite expression data to help understand or rationalize specific metabolic diseases, conditions or biomarkers (through SMP-MAP); 4) determine the similarity of a newly found/synthesized compound to an existing drug (via the ChemQuery search); 5) determine the possible mechanism of action or protein targets for a newly found/synthesized compound (via the ChemQuery search); 6) ascertain whether a certain protein found in bacteria, fungi or viruses could be a drug target (via the SeqSearch query); 7) ascertain whether a newly identified human protein, such as an isoform or paralogue, may be a drug target or a disease indicator (through the SeqSearch query); or 8) use the pathway visualization and mapping tools to explain or teach others about metabolic diseases, basic metabolism or drug action.

## 4. HMDB – A Resource for Biomarker Discovery and Disease Diagnosis

The Human Metabolome Database (HMDB) is the by-product of the Human Metabolome Project – a 3-year (2005–2008), $7.5 million dollar project that was aimed at collating, identifying and annotating all the endogenous metabolites in the human body [38]. The HMDB is actually the largest and most comprehensive, organism-specific metabolomic database assembled to date. It contains spectroscopic, quantitative, analytic and molecular-scale information about human metabolites, their associated enzymes or transporters, their abundance and their disease-related properties. The HMDB currently contains more than 8000 human metabolite entries that are linked to more than 45,000 different synonyms. These metabolites are further connected to 3360 distinct enzymes, which in turn, are linked to nearly 100 metabolic pathways and more than 150 disease pathways. More than 1000 metabolites have disease-associated information, including both normal and abnormal metabolite concentration values. These diagnostic metabolites or metabolite signatures are linked to more than 500 different diseases (genetic and acquired). The HMDB also contains experimental metabolite concentration data for “normal” plasma, urine, CSF and/or other biofluids for more than 5000 compounds. More than 900 compounds are also linked to experimentally acquired “reference” ^1^H and ^13^C NMR and MS/MS spectra. The entire database, including text, sequence, structure and image data occupies nearly 30 Gigabytes of data – most of which can be freely downloaded.

The HMDB is a fully searchable database with many built-in tools for viewing, sorting and extracting metabolites, biofluid concentrations, enzymes, genes, NMR or MS spectra and disease information. As with any web-enabled database, the HMDB supports standard text queries (through the text search box located near the top of each page). It also offers extensive support for higher-level database search and selection functions through a navigation bar (located at the top of each page). The navigation bar has six pull-down menu tabs (“Home”, “Browse”, “Search”, “About”, “Download” and “Contact Us”). The “Browse” tab allows users to select from six browsing options including “HMDB Browse”, “Disease Browse”, “PathBrowse”, “Biofluid Browse”, “HML Browse” and “ClassBrowse”. “HMDB Browse” allows users to search through the HMDB compound by compound through a series of hyperlinked, synoptic summary tables. These metabolite tables can be rapidly browsed, sorted or reformatted in a manner similar to the way PubMed abstracts may be viewed. Clicking on the MetaboCard button found in the leftmost column of any given HMDB summary table opens a webpage describing the compound of interest in much greater detail. Each MetaboCard entry contains more than 100 data fields with half of the information being devoted to chemical or physico-chemical data and the other half devoted to biological or biomedical data. These data fields include a comprehensive compound description, names and synonyms, structural information, physico-chemical data, reference NMR and MS spectra, biofluid concentrations (normal and abnormal), disease associations, pathway information, enzyme data, gene sequence data, protein sequence data, SNP and mutation data as well as extensive links to images, references and other public databases such as KEGG [10], BioCyc [12], PubChem [18], ChEBI [17], PubMed, PDB [7], SwissProt/UniProt [37], GenBank [5], and OMIM [39].

Outside of “HMDB Browse”, there are five other browsing options that allow users to explore or navigate the database. “Disease Browse” allows users to view known metabolic disorders (as well as other diseases) and the metabolites that are typically associated with these conditions. It also allows users to enter lists of metabolites and to identify which diseases are characterized by perturbations to these metabolite levels. “PathBrowse” allows users to browse through the custom-drawn HMDB pathway images. Each pathway is named and each image is zoomable and extensively hyperlinked. Users may also search PathBrowse using lists of compounds (obtained from a metabolomic experiment) and view hyperlinked tables that display all of the pathways that are potentially affected. “Biofluid Browse” allows users to browse metabolite entries based on their concentrations and the biofluids in which they are found. Users may select entries by biofluid type and sort the table by compound name, HMDB ID, concentration, disease, age, or gender. “HML Browse” allows users to browse or search through the Human Metabolome Library (HML). The HML is a library of ∼1000 reference metabolites stored in −80°C freezers at the Human Metabolome Project Centre in Edmonton, Canada. “ClassBrowse”, is designed to allow users to view compounds according to their chemical class designation. Each displayed compound name is hyperlinked to an HMDB MetaboCard. Users may search for compounds (via a text box) or select to view certain compound classes using a pull-down menu located at the top of the ClassBrowse page.

In addition to the data browsing and sorting features already described, the HMDB also offers a chemical structure search utility, a local BLAST search [4] that supports both single and multiple sequence queries, a Boolean text search based on KinoSearch (http://www.rectangular.com/kinosearch/), a chemical structure search utility based on ChemAxon's MarvinView, a relational data extraction tool, an MS spectral matching tool and an NMR spectral search tool (for identifying compounds via MS or NMR data from other metabolomic studies). These can all be accessed via the database navigation bar located at the top of every HMDB page.

HMDB's simple text search supports text matching, text match rankings, mis-spellings (offering suggestions for incorrectly spelled words) and highlights text where the word is found. In addition to this simple text search, HMDB's TextQuery function uses the same KinoSearch engine, but also supports more sophisticated text querying functions (Boolean logic, multi-word matching and parenthetical groupings) as well as data-field-specific queries such as finding the query word only in the “Compound Source” field.

The HMDB's structure similarity search tool (ChemQuery) is the equivalent to BLAST for chemical structures. Users may sketch (through MarvinView's chemical sketching applet) or paste a SMILES string (40) of a query compound into the ChemQuery window. Submitting the query launches a structure similarity search tool that looks for common substructures from the query compound that match the HMDB's metabolite database. High scoring hits are presented in a tabular format with hyperlinks to the corresponding MetaboCards (which in turn links to the protein target). The ChemQuery tool allows users to quickly determine whether their compound of interest is a known metabolite or chemically related to a known metabolite. In addition to these structure similarity searches, the ChemQuery utility also supports compound searches on the basis of chemical formula and molecular weight ranges.

HMDB's BLAST search (SeqSearch) allows users to search through the HMDB via sequence similarity as opposed to chemical similarity. A given gene or protein sequence may be searched against the HMDB's sequence database of metabolically important enzymes and transporters by pasting the FASTA formatted sequence (or sequences) into the SeqSearch query box and pressing the “submit” button. A significant hit reveals, through the associated MetaboCard hyperlink, the name(s) or chemical structure(s) of metabolites that may act on that query protein. With SeqSearch metabolite-protein interactions from model organisms (chimp, rat, mouse, dog, cat, etc.) may be mapped to these organisms via the human data in the HMDB.

The HMDB's data extraction utility (Data Extractor) employs a simple relational database system that allows users to select one or more data fields and to search for ranges, occurrences or partial occurrences of words or numbers. The Data Extractor uses clickable web forms so that users may intuitively construct SQL-like queries. The data extraction tool allows users to easily construct complex queries as “find all diseases where the concentration of homogentisic acid in urine is greater than 1 mM”.

**Figure 2:**
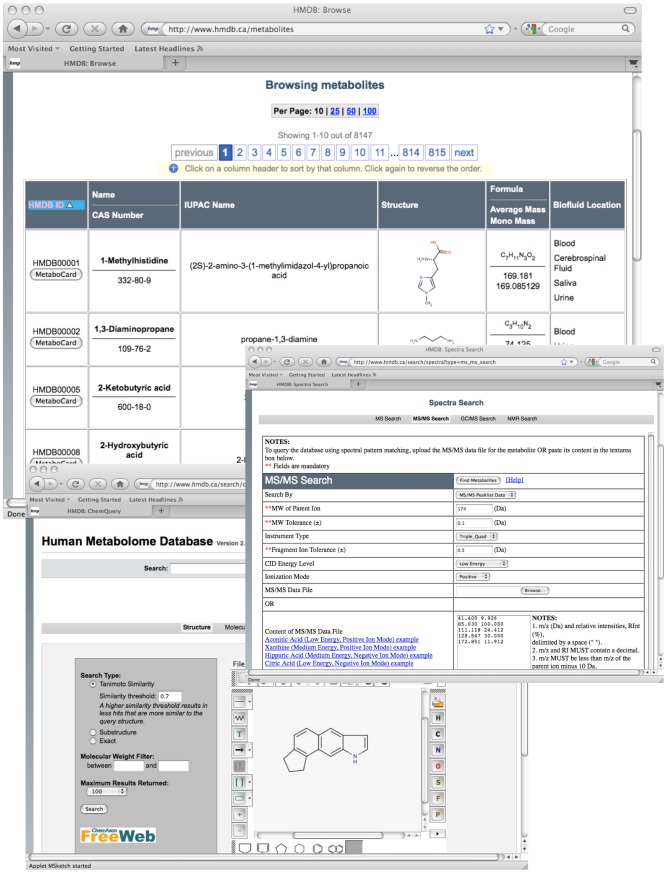
A screenshot montage illustrating the types of viewing and searching options available in HMDB (http://www.hmdb.ca).

The NMR and MS search utilities allow users to upload spectra (for the MS search) or peak lists (for the NMR search) and to search for matching compounds from the HMDB's collection of MS and NMR spectra. In particular, the HMDB contains more than 2000 experimentally collected ^1^H and ^13^C NMR spectra for 900 pure compounds (most collected in water at pH 7.0). It also contains approximately 3800 predicted ^1^H and ^13^C NMR spectra for 1900 other compounds for which authentic samples could not be acquired. The HMDB's mass spectra library contains 2400 MS/MS (Triple-Quad) spectra collected at 3 different collision energies for more than 800 pure compounds. The HMDB's spectral search utilities allow both pure compounds and mixtures of compounds to be identified from their MS or NMR spectra via peak matching algorithms. Compounds may also be identified or searched for by entering their chemical formula or their mass (either their exact mass or a mass range). Figure 2 provides a screenshot montage illustrating the types of viewing and searching options available in HMDB.

To summarize the HMDB allows users to link endogenous metabolites (both their identity and their concentration) to a variety of disease conditions, including metabolic disorders, genetic diseases, chronic (age-related) disorders and a variety of infectious diseases. It also provides links between metabolites and their targets – both through descriptions of the compounds and their known biological roles and through the identification of known pathways or catalyzing enzymes. In addition, the HMDB also supports the direct identification of potential diagnostic biomarkers based on their mass, mass spectra or NMR spectra. Because of this linkage, the HMDB can potentially support a variety of translational bionformatic or cheminformatic queries. For example, through the HMDB it is possible for users to: 1) identify a novel biomarker for a given condition or disease given an NMR or GC/MS or MS/MS spectrum of the purified compound (via the MS/NMR search tools); 2) identify metabolites from a biofluid mixture that has been analyzed by NMR, GC/MS or MS/MS (via the MS/NMR search tools); 3) identify a disease or condition given a list of metabolites (via Disease Browse); 4) identify a pathway or process that has been altered/perturbed given a list of metabolites obtained from a metabolomic experiment (via PathBrowse); 5) determine normal and abnormal concentration ranges for metabolites in different biofluids (via Biofluid Browse); 6) obtain authentic standards of unique metabolites to confirm the diagnosis of a certain disease (via HML Browse); 7) determine the similarity of a newly found/synthesized compound to an existing metabolite (via the structure similarity search); 8) determine the possible mechanism of action or protein targets for a newly discovered/synthesized metabolite or metabolite analogue (via the structure similarity search); 9) diagnose or determine the cause of illnesses thought to be brought on by metabolite changes (through the text search); 10) extract detailed information on metabolites, metabolic diseases or metabolic pathways (via the data extractor); 11) extract information on common metabolite classes (via the data extractor or ClassBrowse); 12) ascertain whether a certain protein or protein homologue may also be involved in a metabolic process or pathway (via the sequence search).

## 5. DrugBank – A Resource for Drug Discovery and Disease Treatment

As previously noted, DrugBank [32] is essentially a hybrid clinically AND chemically oriented drug database that links sequence, structure and mechanistic data about drug molecules with sequence, structure and mechanistic data about their drug targets. DrugBank was one of the first electronic databases to provide the explicit linkage between drugs and drug targets and this particular feature made DrugBank particularly popular. Another important innovation in this database was the presentation of drug and drug target data in synoptic DrugCards (in anology to library cards or study flash-cards). This concept (which is now used in many other chemical-bioinformatic databases) helped make DrugBank particularly easy to view and navigate. Currently DrugBank contains detailed information on 1480 FDA-approved drugs corresponding to 28,447 brand names and synonyms. This collection includes 1281 synthetic small molecule drugs, 128 biotech (mostly peptide or protein) drugs and 71 nutraceutical drugs or supplements. DrugBank also contains information on the 1669 different targets (protein, lipid or DNA molecules) and metabolizing enzymes with which these drugs interact. Additionally the database maintains data on 187 illicit drugs (i.e. those legally banned or selectively banned in most developed nations) and 64 withdrawn drugs (those removed from the market due to safety concerns). Chemical, pharmaceutical and biological information about these classes of drugs is extremely important, not only in understanding their adverse reactions, but also in being able to predict whether a new drug entity may have unexpected chemical or functional similarities to a dangerous or highly addictive drug.

As with the HMDB, the DrugBank website contains many built-in tools and a variety of customized features for viewing, sorting, querying and extracting drug or drug target data. These include a number of higher-level database searching functions such as a local BLAST [4] sequence search (SeqSearch) that supports both single and multiple protein sequence queries (for drug target searching), a boolean text search (TextSearch) for sophisticated text searching and querying, a chemical structure search utility (ChemQuery) for structure matching and structure-based querying as well as a relational data extraction tool (Data Extractor) for performing complex queries.

The BLAST search (SeqSearch) is particularly useful for drug discovery applications as it can potentially allow users to quickly and simply identify drug leads from newly sequenced pathogens. Specifically, a new sequence, a group of sequences or even an entire proteome can be searched against DrugBank's database of known drug target sequences by pasting the FASTA formatted sequence (or sequences) into the SeqSearch query box and pressing the “submit” button. A significant hit can reveal the name(s) or chemical structure(s) of potential drug leads that may act on that query protein (or proteome). The structure similarity search tool (ChemQuery) can be used in a similar manner to SeqSearch. For instance, users may sketch a chemical structure or paste a SMILES string [40] of a possible drug lead or a drug that appears to be causing an adverse reaction into the ChemQuery window. After submitting the query, the database launches a structure similarity search that looks for common substructures from the query compound that match DrugBank's database of known drug or drug-like compounds. High scoring hits are presented in a tabular format with hyperlinks to the corresponding DrugCards. The ChemQuery tool allows users to quickly determine whether their compound of interest acts on the desired protein target or whether the compound of interest may unexpectedly interact with unintended protein targets.

In addition to these search features, DrugBank also provides a number of general browsing tools for exploring the database as well as several specialized browsing tools such as PharmaBrowse and GenoBrowse for more specific tasks. For instance, PharmaBrowse is designed to address the needs of pharmacists, physicians and medicinal chemists who tend to think of drugs in clusters of indications or drug classes. This particular browsing tool provides navigation hyperlinks to more than 70 drug classes, which in turn list the FDA-approved drugs associated with the drugs. Each drug name is then linked to its respective DrugCard. GenoBrowse, on the other hand, is specifically designed to address the needs of geneticists or those specialists interested in specific Drug-SNP relationships. This browsing tool provides navigation hyperlinks to more than 60 different drugs, which in turn list the target genes, SNPs and the physiological effects associated with these drugs.

In addition to its general utility as a general drug encyclopedia, DrugBank also contains several tables, data fields or data types that are particularly useful for pharmacogenomic or pharmacogenetic studies. These include synoptic descriptions of a given drug's Pharmacology as well as its Mechanism of Action, Contraindications, Toxicity, Phase I Metabolizing Enzymes (name, protein sequence and SNPs), and associated Drug Targets (names, protein sequence, DNA sequence, chromosome location, locus number and SNPs). The information contained in DrugBank's Pharmacology, Mechanism of Action, Contraindications and Toxicity fields often includes details about any known adverse reactions. This may include descriptions of known phase I or phase II enzyme interactions, alternate metabolic routes or the existence of secondary drug targets. Secondary drug targets represent proteins (or other macromolecules) that are different than the primary target for which the drug was initially designed or targeted towards. Some drugs may have five or more targets, of which only one might be relevant to treating the disease. DrugBank uses a relatively liberal interpretation of drug targets in order to help identify these secondary drug targets. In particular, for DrugBank a drug target is defined as any macromolecule identified in the literature that binds, transports or transforms a drug. The binding or transformation of a drug by a secondary drug target or an “off-target” protein is one of the most common causes for unwanted side effects or adverse drug reactions (ADRs) [41]. By providing a fairly comprehensive listing of secondary drug targets (along with their SNP information and other genetic data), DrugBank is potentially able to provide additional insight into the underlying causes of a patient's response to a given drug.

DrugBank also provides detailed sequence and SNP data on known drug metabolizing enzymes and known drug targets. In particular DrugBank contains detailed summary tables about each of the SNPs for each of the drug targets or drug metabolizing enzymes that have been characterized by various SNP typing efforts, such as the SNP Consortium [42] and HapMap [43]. Currently DrugBank contains information on 26,292 coding (exon) SNPs and 73,328 non-coding (intron) SNPs derived from known drug targets. It also has data on 1188 coding SNPs and 8931 non-coding SNPs from known drug metabolizing enzymes. By clicking on the “Show SNPs” hyperlink listed beside either the metabolizing enzymes or the drug target SNP field, the SNP summary table can be viewed. These tables include: 1) the reference SNP ID (with a hyperlink to dbSNP); 2) the allele variants; 3) the validation status; 4) the chromosome location and reference base position; 5) the functional class (synonymous, non-synonymous, untranslated, intron, exon); 6) mRNA and protein accession links (if applicable); 7) the reading frame (if applicable); 8) the amino acid change (if existent); 9) the allele frequency as measured in African, European and Asian populations (if available) and 10) the sequence of the gene fragment with the SNP highlighted in a red box.

The purpose of these SNP tables is to allow one to go directly from a drug of interest to a list of potential SNPs that may contribute to the reaction or response seen in a given patient or in a given population. In particular, these SNP lists may serve as hypothesis generators that allow SNP or gene characterization studies to be somewhat more focused or targeted. By comparing the experimentally obtained SNP results to those listed in DrugBank for that drug (and its drug targets) it may be possible to ascertain which polymorphism for which drug target or drug metabolizing enzyme may be contributing to an unusual drug response. Obviously these database-derived SNP suggestions may require additional experimental validation to prove their causal association.

**Figure 3:**
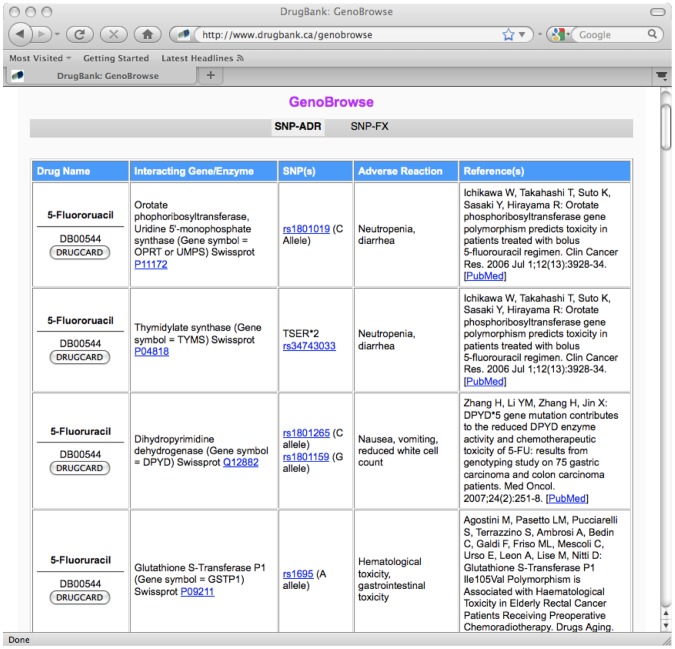
A screen shot of DrugBank's SNP-ADR table. This displays the information on the adverse drug reactions (ADRs) and associated SNP (single nucleotide polymorphisms) with certain drugs and drug targets (http://www.drugbank.ca).

Drugbank also includes two tables that provide much more explicit information on the relationship between drug responses/reactions and gene variant or SNP data. The two tables, which are accessible from the GenoBrowse submenu located on DrugBank's Browse menu bar, are called SNP-FX (short for SNP-associated effects) and SNP-ADR (short for SNP-associated adverse drug reactions). SNP-FX contains data on the drug, the interacting protein(s), the “causal” SNPs or genetic variants for that gene/protein, the therapeutic response or effects caused by the SNP-drug interaction (improved or diminished response, changed dosing requirements, etc.) and the associated references describing these effects in more detail. SNP-ADR follows a similar format to SNP-FX but the clinical responses are restricted only to adverse drug reactions (ADR). SNP-FX contains literature-derived data on the therapeutic effects or therapeutic responses for more than 70 drug-polymorphism combinations, while SNP-ADR contains data on adverse reactions compiled from more than 50 drug-polymorphsim pairings. All of the data in these tables is hyperlinked to drug entries from DrugBank, protein data from SwissProt, SNP data from dbSNP and bibliographic data from PubMed. A screen shot of the SNP-ADR table is shown in Figure 3. As can be seen from the figure, these tables provide consolidated, detailed and easily accessed information that clearly identifies those SNPs that are known to affect a given drug's efficacy, toxicity or metabolism.

To summarize, DrugBank allows users to link drugs to a variety of disease conditions or health indications. It also provides links between drugs and their targets – both through descriptions of the mechanism of action and through the identification of known protein (or gene) targets. Because of this kind of extensive data linkage, DrugBank can potentially support a number of translational bionformatic or cheminformatic questions. For example, through DrugBank it is possible for users to: 1) determine the similarity of a newly found/synthesized compound to an existing drug (via the structure similarity search); 2) determine the possible mechanism of action or protein targets for a newly found/synthesized compound (via the structure similarity search); 3) diagnose or determine the cause of illnesses thought to be brought on by adverse drug reactions (through the text search or SNPADR/SNPFX); 4) treat or find references to the treatment of illnesses based on symptoms or disease diagnosis (via the text search); 5) extract information on common drug targets (via the data extractor or the sequence search); 6) extract information on common drug classes or structures (via the data extractor or the structure search); 7) ascertain whether a certain protein found in bacteria, fungi or viruses could be a drug target (via the sequence search); or 8) ascertain whether a newly identified human protein, such as an isoform or paralogue, may be a drug target (through the sequence search).

## 6. T3DB – A Resource linking Small Molecules to Disease & Toxicity

A toxic substance is a small molecule, peptide, or protein that is capable of causing injury, disease, genetic mutations, birth defects or death. Toxins, both natural and man-made, represent an important class of poisonous compounds that are ubiquitous in nature, in homes, and in the workplace. Common toxins include pollutants, pesticides, preservatives, drugs, venoms, food toxins, cosmetic toxins, dyes, and cleaning compounds. Because toxic compounds are essentially disease-causing agents, it has long been recognized that there is a need to associate toxic compound data with molecular toxicology and clinical symptomology. While this has been done in a variety of toxicology textbooks and medical reference manuals, it has only recently been done using electronic databases and the tools associated with bioinformatics and cheminformatics.

T3DB [36] is currently the only chemical-bioinformatic database that provides in-depth, molecular-scale information about toxins, their associated targets, their toxicology, their toxic effects and their potential treatments. T3DB currently contains over 3000 toxic substance entries corresponding to more than 34,000 different synonyms. These toxins are further connected to some 1450 protein targets through almost 35,500 toxin and toxin-target associations. These associations are supported by more than 5400 references. The entire database, including text, sequence, structure and image data, occupies nearly 16 Gigabytes of data – most of which can be freely downloaded.

**Figure 4:**
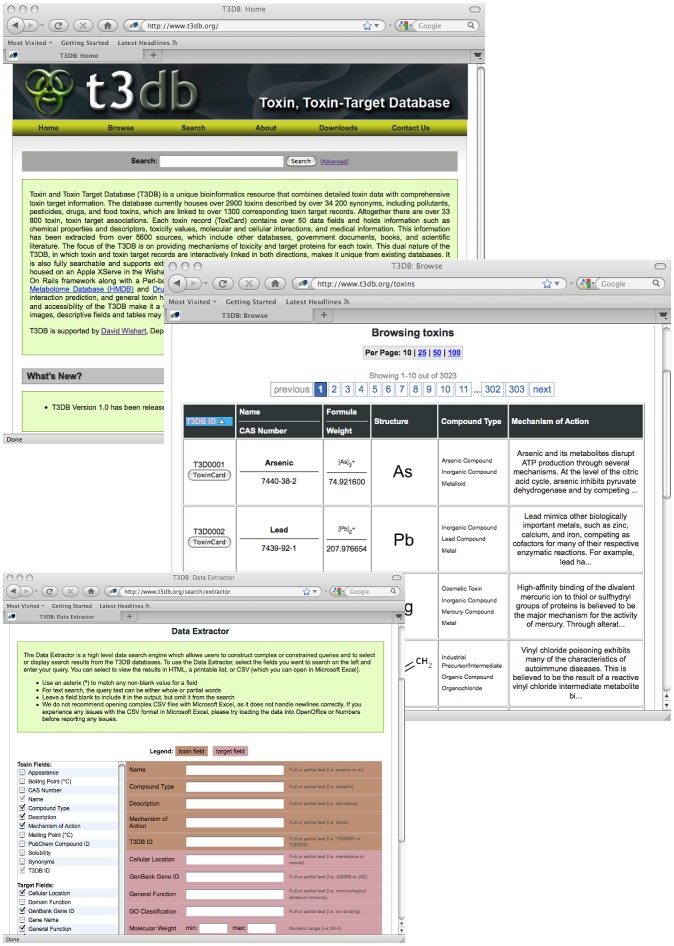
A screenshot montage illustrating the types of viewing and searching options available in T3DB (http://www.t3db.org).

As with HMDB and DrugBank, the T3DB is designed to be a fully searchable web resource with many built-in tools and features for viewing, sorting and extracting toxin and toxin-target annotation, including structures and gene and protein sequences. A screenshot montage illustrating the types of viewing and searching options available is shown in Figure 4. As with HMDB and DrugBank, the T3DB supports standard text queries through the text search box located on the home page. It also offers general database browsing using the “Browse” button located in the T3DB navigation bar. To facilitate browsing, the T3DB is divided into synoptic summary tables which, in turn, are linked to more detailed “ToxCards”- in analogy to the DrugCard concept found in DrugBank [32] or the MetaboCard in HMDB [26]. All of the T3DB's summary tables can be rapidly browsed, sorted or reformatted in a manner similar to the way PubMed abstracts may be viewed. Clicking on the ToxCard button, found in the leftmost column of any given T3DB summary table, opens a webpage describing the toxin of interest in much greater detail. Each ToxCard entry contains over 80 data fields, with ∼50 data fields devoted to chemical and toxicological/medical data and ∼30 data fields (each) devoted to describing the toxin target(s).

A ToxCard begins with various identifiers and descriptors (names, synonyms, compound description, structure image, related database links and ID numbers), followed by additional structure and physico-chemical property information. The remainder of data on the toxin is devoted to providing detailed toxicity and toxicological data, including route of delivery, mechanism of action, medical information, and toxicity measurements. All of a toxin's targets are also listed within the ToxCard. Each of these targets are described by some 30 data fields that include both chemical and biological (sequence, molecular weight, gene ontology terms, etc.) information, as well as details on their role in the mechanism of action of the toxin. In addition to providing comprehensive numeric, sequence and textual data, each ToxCard also contains hyperlinks to other databases, abstracts, digital images and interactive applets for viewing the molecular structures of each toxic substance.

A key feature that distinguishes the T3DB from other on-line toxin or toxicology resources is its extensive support for higher-level database search and selection functions. In addition to the data viewing and sorting features already mentioned, the T3DB also offers a local BLAST search that supports both single and multiple sequence queries, a boolean text search based on KinoSearch, a chemical structure search utility based on ChemAxon's MarvinView, and a relational data extraction tool similar to that found in DrugBank and the HMDB [26], [32]. These can all be accessed via the database navigation bar located at the top of every T3DB page.

T3DB's simple text search box (located at the top of most T3DB pages) supports text matching, text match rankings, mis-spellings and highlights text where the word is found. In addition to this simple text search, T3DB's TextQuery function supports more sophisticated text querying functions including “and” and “or” queries, multi-word matching and parenthetical groupings as well as data-field-specific queries such as finding the query word only in the “Compound Source” field. Additional details and examples are provided on the T3DB's TextQuery page.

T3DB's sequence searching utility (SeqSearch) allows users to search through T3DB's collection of 1450 known (human) toxin targets. This service potentially allows users to identify both orthologous and paralogous targets for known toxins or toxin targets. It also facilitates the identification of potential toxin targets from other animal species. With SeqSearch, gene or protein sequences may be searched against the T3DB's sequence database of identified toxin-target sequences by pasting the FASTA formatted sequence (or sequences) into the SeqSearch query box and pressing the “submit” button.

T3DB's structure similarity search tool (ChemQuery) can be used in a similar manner as its SeqSearch tool. Users may sketch a chemical structure (through ChemAxon's freely available chemical sketching applet) or paste a SMILES string of a query compound into the ChemQuery window. Submitting the query launches a structure similarity search that looks for common substructures from the query compound that matches the T3DB's database of known toxic compounds. Users can also select the type of search (exact or Tanimoto score) to be performed. High scoring hits are presented in a tabular format with hyperlinks to the corresponding ToxCards (which, in turn, links to the targets). The ChemQuery tool allows users to quickly determine whether their compound of interest is a known toxin or chemically related to a known toxin and which target(s) it may act upon. In addition to these structure similarity searches, the ChemQuery utility also supports compound searches on the basis of SMILES strings (under the SMILES tab) and molecular weight ranges (under the Molecular Weight tab).

The T3DB's data extraction utility (Data Extractor) employs a simple relational database system that allows users to select one or more data fields and to search for ranges, occurrences or partial occurrences of words, strings, or numbers. The data extractor uses clickable web forms so that users may intuitively construct SQL-like queries. Using a few mouse clicks, it is relatively simple to construct complex queries (“find all toxins that target acetylcholinesterase and are pesticides”) or to build a series of highly customized tables. The output from these queries is provided in HTML format with hyperlinks to all associated ToxCards.

To summarize, T3DB allows users to link toxic substances to a variety of disease conditions, including acute toxicity, long-term toxicity, birth defects, cancer, other illnesses. It also provides links between toxic substances and their targets – both through descriptions of the mechanism of action and through the identification of known protein (or gene) targets. Because of this kind of extensive data linkage, T3DB can potentially support a variety of bioinformatic or cheminformatic queries. For example, through T3DB it is possible for users to: 1) determine the similarity of a newly found/synthesized compound to an existing toxin (via the structure similarity search); 2) determine the possible mechanism of action or protein targets for a newly found/synthesized compound (via the structure similarity search); 3) diagnose or determine the cause of illnesses thought to be brought on by exposure to a given toxin (through the text search); 4) treat or find references to the treatment of illnesses brought on by exposure to a given toxin (via the text search); 5) extract information on common toxin targets (via the data extractor); 6) extract information on common toxin classes (via the data extractor); 7) ascertain whether a certain protein or protein homologue may also be a toxin target (via the sequence search); or 8) ascertain whether a newly identified peptide or protein may be a toxin (through the sequence search).

## 7. Software for Interpreting Small Molecule and Disease Data

With the recent emergence of chemical-bioinformatic databases having a solid translational (i.e. biomedical) functionality, the way has been cleared for the development of software tools that exploit these databases. This is a natural process in both bioinformatics and cheminformatics as databases always appear before any software applications are typically developed. Given that the field of chemical bioinformatics is still quite young and the number of databases with disease and small molecule information is still relatively small, it is not surprising to find that the number of software tools developed to exploit these databases is still quite small. Here we will briefly describe two recently developed software tools – PolySearch and MSEA – that exploit the data in SMPDB, HMDB and DrugBank to perform a number of useful applications.

### 7.1 Text Mining with PolySearch

PolySearch [44] is a freely available, web-based text-mining tool that allows users to search through large numbers of PubMed abstracts to make large-scale linkages or associations. Examples of large-scale associations are: “Find all genes associated with breast cancer” or “Find all diseases treatable by tamoxifen”. In order to conduct the first query using PubMed, one would have to have a list of all known human genes and perform 25,000+ queries with each gene name and the words “breast cancer”. To conduct the second query, it would be necessary to have a list of all known diseases (more than 5000 are known) and perform 5000+ queries with the word “tamoxifen” included in each query. Obviously this would take a person a very long time. However, using a computer to perform these repeated queries would be much less tedious and much faster. PolySearch is designed to rapidly perform these types of expansive queries by exploiting the PubMed application programming interface (API) and a special collection of dictionaries and thesauruses compiled from various bioinformatic and chemical-bioinformatic databases. In particular, the typical query supported by PolySearch is “Given X, find all Y's” where X or Y can be diseases, tissues, cell compartments, gene/protein names, SNPs, mutations, drugs and metabolites. The disease names and synonyms in PolySearch are derived from medical dictionaries and MeSH (medical subject headings), gene and protein names/synonyms are derived from UniProt, drug names/synonyms are derived from DrugBank while metabolites and metabolite synonyms are derived from the HMDB. Obviously, without these small molecule dictionaries or thesauruses, many of PolySearch's queries could not be performed.

PolySearch also exploits a variety of techniques in text mining and information retrieval to identify, highlight and rank informative abstracts, paragraphs or sentences. A central premise to PolySearch's search strategy is the assumption that the greater the frequency with which an X and Y association occurs within a collection of abstracts, the more significant the association is likely to be. For instance, if COX2 is mentioned in PubMed as being associated with colon cancer 510 times but thioredoxin is associated with colon cancer only once, then one is more likely to have more confidence in the COX2-colon cancer association. Frequency alone is not always the best way to rate a paper or a website for its relevancy. Therefore, in addition to counting the frequency of apparent associations, PolySearch employs a specially developed text-ranking scheme to score the most relevant sentences and abstracts that associate both the query and match terms with each other.

In summary, PolySearch is able to exploit the name and synonym sets from a number of small-molecule and disease databases (HMDB, DrugBank, MeSH, OMIM) thereby allowing users to perform a range of text mining queries on the PubMed abstract database. In particular, PolySearch allows users to find newly described or previously unknown (to the user, at least) associations between: 1) drugs and disease; 2) metabolites and disease; 3) genes/proteins and disease; 4) drugs and drug targets; 5) metabolites and metabolizing enzymes; 6) SNPs and disease and 7) mutations and disease. In addition, through its other query fields or query options, PolySearch is able to perform a large number (>50) of other text queries that may be relevant to a variety of applications in translational bioinformatics.

### 7.2 Metabolite Set Enrichment Analysis

The Metabolite Set Enrichment Analysis (MSEA) server [45] is a web-based tool designed to help researchers identify and interpret patterns of human or mammalian metabolite concentration changes in a biologically meaningful context. It is based on the concepts originally developed for gene expression or microarray analysis called Gene Set Enrichment Analysis or GSEA [8]. The central idea behind GSEA is to directly investigate the enrichment of pre-defined groups of functionally related genes (or gene sets) instead of individual genes. This group-based approach does not require pre-selection of genes with an arbitrary threshold. Instead, functionally related genes are evaluated together as gene sets, allowing additional biological information to be incorporated into the analysis process. Key to the development of GSEA has been the compilation of libraries or databases of gene expression changes that are associated with specific conditions, pathways, diseases or perturbations. Therefore in order to develop MSEA, it was necessary to extract a large body of metabolite expression changes (i.e. chemical profiles) and metabolic pathway information from a variety of databases. Fortunately, the existence of SMPDB and HMDB made the compilation of this metabolite expression library relatively easy. By downloading the freely available data in HMDB and SMPDB, the authors of MSEA were able to construct a collection of five metabolite set libraries containing ∼1,000 biologically meaningful groups of metabolites. In MSEA, a group of metabolites are considered to constitute a metabolite set if they are known to be: a) involved in the same biological processes (i.e., metabolic pathways, signaling pathways); b) changed significantly under the same pathological conditions (i.e., various metabolic diseases); and c) present in the same locations such as organs, tissues or cellular organelles. The resulting metabolite sets were organized into three categories: pathway-associated, disease-associated, and location based. MSEA's pathway-associated metabolite library contains 84 entries based on the 84 human metabolic pathways found in SMPDB. MSEA's disease-associated metabolite sets were mainly collected from information in the HMDB, the Metabolic Information Center (MIC), and SMPDB. Using these resources, a total of 851 physiologically informative metabolite sets were created. These disease-associated metabolite sets were further divided into three sub-categories based on the biofluids in which they were measured: 398 metabolite sets in blood, 335 in urine, and 118 in cerebral-spinal fluid (CSF). MSEA's location-based library contains 57 metabolite sets based on the “Cellular Location” and “Tissue Location” listed in the HMDB.

While the exact statistical or enrichment analysis methods used in MSEA are well beyond the scope of this chapter, suffice it to say that MSEA essentially allows one to take lists of metabolites and to identify which pathways, diseases or medical conditions are most likely to be associated with that metabolite set. It is also possible to do the same kind of operation with a list of metabolites and their absolute (or relative) concentrations. While disease/metabolite associations can be made through HMDB and SMPDB, these primitive search tools do not have the same statistical rigor that characterizes a full-fledged enrichment analysis. Furthermore, the MSEA pathway and disease data set is somewhat larger than what is found in the HMDB or SMPDB. This means that MSEA will be far more likely to find a useful (and statistically significant) pathway or disease than what could be done with HMDB or SMPDB.

Overall, MSEA is an example of an analytical software tool that exploits chemical-bioinformatic data to perform robust statistical analyses of metabolomic or clinical chemistry data. Given their close similarity, it is reasonable to expect MSEA could eventually be integrated with GSEA, thereby allowing a comprehensive analysis of both gene and metabolite expression changes on a single integrated program or website. No doubt this kind of integrated “omic” analysis tool is not far away from being developed.

## 8. Summary

With today's focus on genes and proteins as the “primary” causes or biomarkers of disease, the relationship between small molecules and human disease is often overlooked. However it is important to remember that more than 95% of all diagnostic clinical assays are designed to detect small molecules (i.e. blood glucose, serum creatinine, amino acid analysis, etc.). Likewise nearly 90% of all known drugs are small molecules, 50% of all drugs are derived from pre-existing metabolites and 30% of identified genetic disorders involve diseases of small molecule metabolism. Clearly, small molecules are important and given the rapid growth in metabolomics, pharmacogenomics and systems biology, it is likely that their role in disease diagnosis and disease treatment will continue to grow. Given these exciting growth prospects and given the importance of small molecules in medicine and translational research, scientists are now realizing that there is a critical need to link information about small molecules to their corresponding “big molecule” targets. This has led to the emergence of a new field of bioinformatics – called chemical bioinformatics.

This chapter has covered several topics related to chemical bioinformatics and the role that chemical bioinformatics can play in identifying the chemicals that cause (toxins), cure (drugs) or characterize (biomarkers) many human diseases. The first part of the chapter gave a brief overview of some of the most important or widely used chemical bioinformatic resources along with a more detailed discussion of some of the major classes of chemical-bioinformatic databases. In particular four major database classes were described: 1) small molecule (or metabolic) pathway databases; 2) metabolite or metabolomic databases; 3) drug databases; and 4) toxin or toxic substance databases. Examples of each of these databases were given and many of their strengths and limitations were discussed. While most of these chemical-bioinformatic databases provide links between small molecules and their large molecule targets, relatively few provide linkages to clinical, physiological or disease information.

The second part of this chapter focused on describing a number of recently developed databases that explicitly relate small molecules to disease. This included detailed descriptions of four databases: 1) the Small Molecule Pathway Database (SMPDB); 2) the Human Metabolome Database (HMDB); 3) DrugBank and 4) the Toxin, Toxin-Target Database (T3DB). SMPDB is a graphically oriented pathway database that contains ∼450 metabolic pathways, disease pathways and drug pathways. The HMDB is a comprehensive metabolomic database that is primarily oriented to answering questions in clinical metabolomic and clinical biochemistry. DrugBank is a comprehensive drug database containing detailed information about drugs, drug targets and clinical pharmacology. The T3DB is a toxicology database containing detailed information about toxins, toxin targets and their corresponding toxicological information. Each of these databases was described in terms of its content, general design and query/search functions. Additionally, explicit examples of various translational or disease-related applications were provided for each database. The final part of this chapter provided a short discussion of some of the newly emerging software tools that exploit these databases, including PolySearch and MSEA (Metabolite Set Enrichment Analysis). PolySearch is a text-mining tool that exploits the synonym data found in these small molecule databases to allow expansive PubMed queries to be performed. MSEA is a metabolomic analysis tool that exploits the pathway and disease information found in SMPDB and HMDB to perform pathway and disease identification from raw metabolomic data.

While the sub-discipline of chemical bioinformatics is still quite young, and the number of tools for translational applications is still relatively small, it should be clear that what is now out there has considerable potential for a wide range of clinical, biomedical, pharmaceutical and toxicological applications. Certainly as more tools are developed and as more databases evolve, it is likely that chemical bioinformatics will soon be able to establish itself as one of the most medically useful sub-disciplines in the entire field of bioinformatics.

## 9. Exercises

1) A compound with a molecular weight of 136.053 daltons has been isolated from the urine of a 3 month-old baby with unusually light coloring of the skin, eczema (an itchy skin rash), and a musty body odor. What compound is it and what disease might this baby have?

2) Your natural product chemist neighbor has just isolated a compound from the Tanzanian periwinkle – a rare plant species found only in the highlands of Eastern Tanzania. Locals use the plant as a treatment for a variety of blood disorders. The structure of the compound is given by the following SMILES string: COC1 = CC = C2C( = CC1 = O)C(CCC1 = CC(OC) = C(OC)C(OC) = C21)NC(CO)

What compound is this similar to, what diseases could it be used to treat and what proteins might it bind?

3) A viral protein with the following sequence has been isolated from a number of dead and dying African Green Monkeys that were housed at a local zoo.

PQVTLYQRPLVTIRVGGQLKEALIDTGADDTVLENMNLPGRWKPKMIGAIAGFIKVKQYDQITVEICGHKGIGTILVGPTPVNIIGRNLLTLIGCTLNF

The illness seems to be spreading to other monkey colonies in the zoo. What drugs could be used to treat the sick monkeys and to prevent the spread of the disease?

4) A farmer who has just finished harvesting his barley field has come into the clinic complaining of skin irritation, burning and itching, a rash and a series of skin blisters. He also has eye pain, conjunctivitis, burning sensations about the eyes, and blurred vision. Other symptoms have included nausea, vomiting and fatigue. Suspecting that he may have been exposed to some toxin or pesticide a chemical analysis has been performed of his blood, urine and lacrimal (tear) fluid. MS analysis of all three fluids has identified an unusual compound with a molecular weight of 296.126 daltons. What compound might this be?

5) What kind of drugs can be used to treat breast cancer? Describe your search strategy and your rationale for this search strategy.

Answers to the Exercises can be found in Text S1.

**Further Reading**

- Villas-Boas SG, Nielson J, Smedsgaard J, Hansen MAE, Roessner-Tunali U, editors (2007) Metabolome analysis: an introduction. New York: John Wiley & Sons.
- Wishart DS (2008) DrugBank and its relevance to pharmacogenomics. Pharmacogenomics 9: 1155–1162.
- Krawetz S, editor (2009) Bioinformatics for systems biology. Totowa: Humana Press.
- Wishart DS (2008) Applications of metabolomics in drug discovery and development. Drugs R D 9: 307–322.
- Baxevanis A (2003) Current protocols in bioinformatics. New York: John Wiley & Sons – see Chapter 14.

**Glossary**

Cheminformatics – a field of information technology that uses computers to facilitate the collection, storage, analysis and manipulation of large quantities of chemical data.

DrugBank – A database containing chemical and biological data on drugs and drug targets.

GSEA – Gene Set Enrichment Analysis. GSEA is a statistically based bioinformatic method designed to directly investigate the enrichment of pre-defined groups of functionally related genes (or gene sets) from gene expression data.

HMDB – The Human Metabolome Database. A database containing chemical and biological data on human metabolites aimed at clinical metabolomic studies.

MS – Mass Spectrometry. An analytical method that measures molecular weight of compounds based on their mass to charge ratio. Mass spectrometry is one of the standard methods to determine the molecular formula of new compounds and to confirm the identity of synthesized chemicals or natural products.

Metabolome – the collection of all small molecule metabolites found in a given cell, tissue, organ or organism.

Metabolomics - a branch of “omics” research that is primarily concerned with the high-throughput identification and quantification of small molecule (<1500 Da) metabolites in the metabolome.

MSEA – Metabolite Set Enrichment Analysis. MSEA is a statistically based bioinformatic method designed to directly investigate the enrichment of pre-defined groups of functionally related metabolites (or metabolite sets) from metabolomic data.

NMR – Nuclear Magnetic Resonance Spectroscopy. An analytical method that measures nuclear magnetism under very high magnetic fields. NMR is the standard method used by chemists today to identify and characterize small molecules.

Pharmacogenomics – A newly emerging field of pharmacology that integrates genotyping and gene expression data with classical pharmacological and adverse drug reaction studies.

SMPDB – The Small Molecule Pathway Database. A database containing pathway diagrams and interactive viewing tools for small molecules involved in metabolism, drug action and disease.

T3DB – The Toxin, Toxin-Target Database. A database with chemical and biological data on common toxins, poisons, household chemicals, pollutants and other harmful substances.

Toxicogenomics – A newly emerging field of toxicology that integrates genotyping and gene expression data with classical toxicological and toxicity studies.

## Supporting Information

Text S1 Answers to Exercises.
(DOC)
