# Supplementary material for: Chapter 3: Small Molecules and Disease
Source: PLoS Comput Biol. 2012 Dec 27;8(12):e1002805. doi: 10.1371/journal.pcbi.1002805 (PMC3531289; doi:10.1371/journal.pcbi.1002805)
Supplement: Text S1 — Answers to Exercises. (DOC) [file pcbi.1002805.s001.doc]

**Exercises**

1) A compound with a molecular weight of 136.053 daltons has been isolated from the urine of a 3 month-old baby with unusually light coloring of the skin, eczema (an itchy skin rash), and a musty body odor. What compound is it and what disease might this baby have?

*Answer: Use the MS search routine in HMDB. The compound will be identified as phenylacetic acid. The disease is phenylketonuria.*

2) Your natural product chemist neighbor has just isolated a compound from the Tanzanian periwinkle – a rare plant species found only in the highlands of Eastern Tanzania. Locals use the plant as a treatment for a variety of blood disorders. The structure of the compound is given by the following SMILES string: COC1=CC=C2C(=CC1=O)C(CCC1=CC(OC)=C(OC)C(OC)=C21)NC(CO)

What compound is this similar to, what diseases could it be used to treat and what proteins might it bind?

*Answer: Use the ChemQuery search in DrugBank using either the structure or SMILES string search. The compound is most similar to Colchicine. Reading through the DrugBank entry for Colchicine one will find that it is known to bind to tubulin proteins and it can be used for treatment of gout and familial Mediterranean fever; it can also be used as initial treatment for pericarditis. It also has anti-cancer properties.*

3) A viral protein with the following sequence has been isolated from a number of dead and dying African Green Monkeys that were housed at a local zoo.

PQVTLYQRPLVTIRVGGQLKEALIDTGADDTVLENMNLPGRWKPKMIGAIAGFIKVKQYDQITVEICGHKGIGTILVGPTPVNIIGRNLLTLIGCTLNF

The illness seems to be spreading to other monkey colonies in the zoo. What drugs could be used to treat the sick monkeys and to prevent the spread of the disease?

*Answer: Using the Sequence Search tool in DrugBank you will find that this protein is very similar to the HIV protease and that the following drugs (using Drugbank IDs) bind to or inhibit HIV-like proteases: DB00220; DB00224; DB00503; DB00701; DB00932; DB01072; DB01232; DB01264; DB01319; DB01601*

4) A farmer who has just finished harvesting his barley field has come into the clinic complaining of skin irritation, burning and itching, a rash and a series of skin blisters. He also has eye pain, conjunctivitis, burning sensations about the eyes, and blurred vision. Other symptoms have included nausea, vomiting and fatigue. Suspecting that he may have been exposed to some toxin or pesticide a chemical analysis has been performed of his blood, urine and lacrimal (tear) fluid. MS analysis of all three fluids has identified an unusual compound with a molecular weight of 296.126 daltons. What compound might this be?

*Answer: Using either the Chem Query (Molecular Weight) or Data Extractor tool in T3DB you will find that the compound is likely vomitoxin and that the symptoms are characteristic of mycotoxin poisoning or acute mycotoxin exposure.*

5) What kind of drugs can be used to treat breast cancer? Describe your search strategy and your rationale for this search strategy.

*Answer: You can use either PolySearch or DrugBank to find the answer. The list of therapies or potential therapies is quite long and won’t be reproduced here.*
